# Supplementary material for: Genome-Wide Analysis of KAP1 Binding Suggests Autoregulation of KRAB-ZNFs
Source: PLoS Genet. 2007 Jun 1;3(6):e89. doi: 10.1371/journal.pgen.0030089 (PMC1885280; doi:10.1371/journal.pgen.0030089)
Supplement: Supplementary file 1 [file pgen.0030089.sg001.pdf]

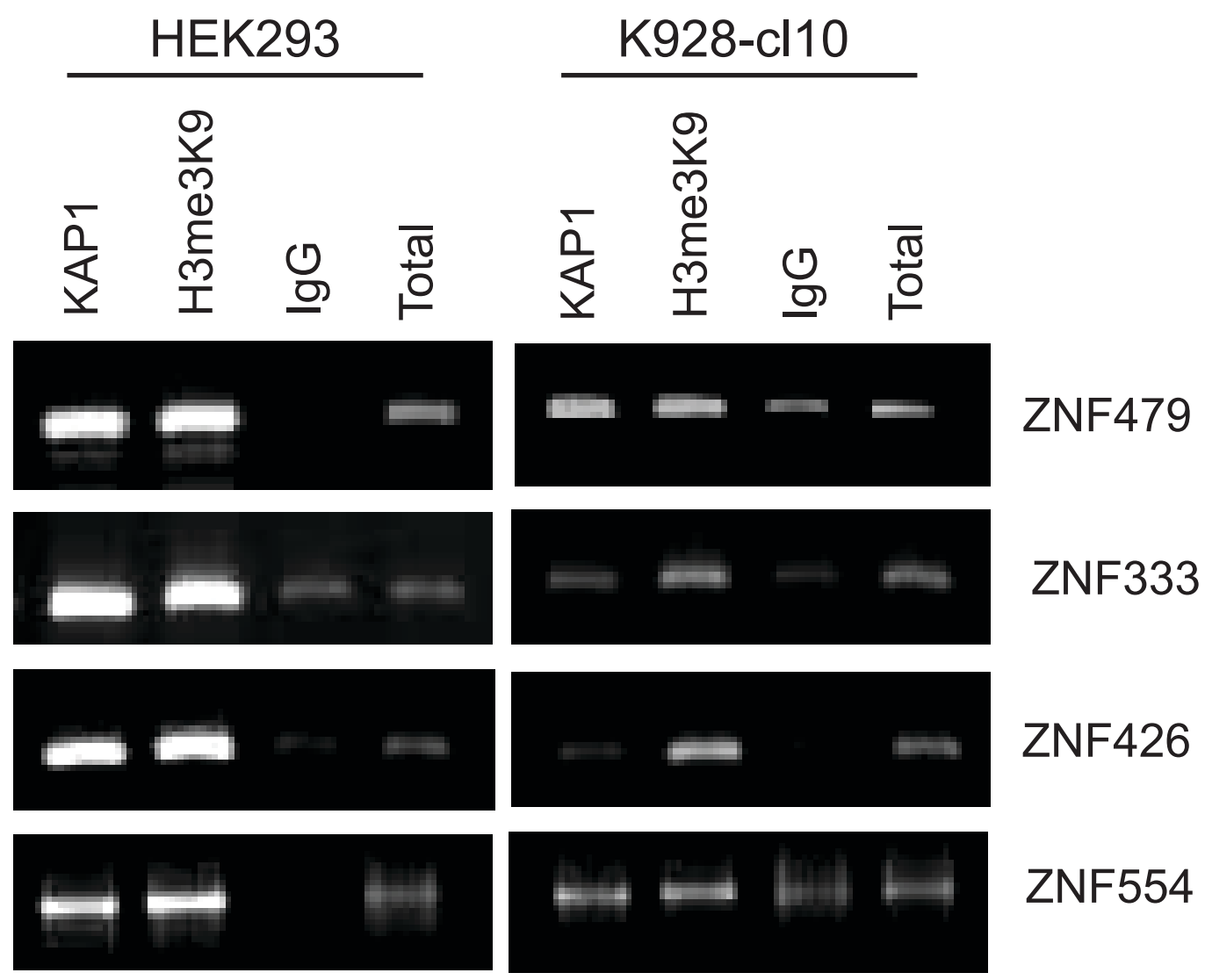

Figure S1. PCR analysis of KAP1 and H3me3K9 amplicons from parent HEK293 cells and the stable KAP1 knockdown cell line K928-cl10 (Sripathy et al., Mol Cell Biol 26: 8623-8638, 2006). The regions examined were identified as KAP1 targets in ChIP-chip assays using Ntera2 cells (see Fig 6B). We found that KAP1 and H3me3K9 were also bound to these targets in HEK293 cells (left panel). As expected, KAP1 binding to the target sites is reduced in the stable KAP1 knockdown cell line. Importantly, H3me3K9 binding is also significantly reduced at the target sites (right panel). In these analyses, signals higher than total indicate that KAP1 and H3me3K9 were bound to the tested sites whereas signals equal to or lower than total are expected if the site is not immunoprecipitated by the antibody. IgG amplicons were also included as a negative control.
